# Plasmatic Membrane Expression of Adhesion Molecules in Human Cardiac Progenitor/Stem Cells Might Explain Their Superior Cell Engraftment after Cell Transplantation

**DOI:** 10.1155/2020/8872009

**Published:** 2020-10-10

**Authors:** Imelda Ontoria-Oviedo, Itziar Palacios, Joaquín Panadero, Belén Sánchez, Francisco García-García, Adolfo López-Cerdán, Akaitz Dorronsoro, Delia Castellano, Luis Rodríguez-Borlado, Antonio Bernad, Pilar Sepúlveda

**Affiliations:** ^1^Regenerative Medicine and Heart Transplantation Unit, Instituto de Investigación Sanitaria La Fe, 46026 Valencia, Spain; ^2^Coretherapix, SLU, Tres Cantos, 28760 Madrid, Spain; ^3^IGENOMIX S.L, 46980 Paterna, Valencia, Spain; ^4^Bioinformatics and Biostatistics Unit, Centro de Investigación Príncipe Felipe, 46012 Valencia, Spain; ^5^Biomedical Imaging Unit FISABIO-CIPF, Centro de Investigación Príncipe Felipe, 46012 Valencia, Spain; ^6^Department of Immunology and Oncology, Centro Nacional de Biotecnología (CNB-CSIC), 28048 Madrid, Spain

## Abstract

Human bone marrow mesenchymal stem cells (BM-MSCs) and cardiac progenitor/stem cells (CPCs) have been extensively studied as a potential therapeutic treatment for myocardial infarction (MI). Previous reports suggest that lower doses of CPCs are needed to improve cardiac function relative to their bone marrow counterparts. Here, we confirmed this observations and investigated the surface protein expression profile that might explain this effect. Myocardial infarction was performed in nude rats by permanent ligation of the left coronary artery. Cardiac function and infarct size before and after cell transplantation were evaluated by echocardiography and morphometry, respectively. The CPC and BM-MSC receptome were analyzed by proteomic analysis of biotin-labeled surface proteins. Rats transplanted with CPCs showed a greater improvement in cardiac function after MI than those transplanted with BM-MSCs, and this was associated with a smaller infarct size. Analysis of the receptome of CPCs and BM-MSCs showed that gene ontology biological processes and KEGG pathways associated with adhesion mechanisms were upregulated in CPCs compared with BM-MSCs. Moreover, the membrane protein interactome in CPCs showed a strong relationship with biological processes related to cell adhesion whereas the BM-MSCs interactome was more related to immune regulation processes. We conclude that the stronger capacity of CPCs over BM-MSCs to engraft in the infarcted area is likely linked to a more pronounced cell adhesion expression program.

## 1. Introduction

Stem cell therapies have emerged as a promising treatment for different pathologies, including cardiovascular diseases, and may pave the way for effective approaches to regenerate the heart and restore cardiac function after injury [1]. In this line, cardiac progenitor/stem cells (CPCs) have been proposed and tested for their participation in cardiac homeostasis and repair [2–6]. Initial clinical trials of autologous cell-based therapy demonstrated the feasibility of CPCs to promote cardiac repair after myocardial infarction (MI) [7, 8], and later studies tested their efficacy in the allogeneic setting [9, 10].

Bone marrow mesenchymal stem cells (BM-MSCs) have also been demonstrated to promote cardiac repair after acute MI (AMI), by attenuating left ventricular remodeling and promoting neoangiogenesis [11, 12]. These effects are primarily ascribed to the ability of BM-MSCs to migrate to damaged or malfunctioning tissues [13, 14] and secrete trophic factors or extracellular vesicles [15] and their potential to suppress immune reactions [16]. Nevertheless, despite the successful results in animal models, the results in human trials have been for the most part disappointing [17–20], which has motivated a reappraisal of their clinical significance. While the mode of action of CPCs and BM-MSCs in cardiac repair is still somewhat unclear, there is a consensus that both types of administered cells release growth factors and molecules that promote angiogenesis and immune regulation, limiting the postinfarct scar, preventing myocardial apoptosis, and stimulating resident CPCs to repair the damage, the so-called paracrine effect [21–23]. It is widely accepted that the immune response triggered after MI plays an important role in the extension of the damage after the ischemic injury and also on disease progression [24, 25]. In that sense, it was suggested that the interaction of the administered cells with cell populations present in the heart after AMI mediates a beneficial effect on inflammation and tissue regeneration [5]. This is supported by the findings that while CPCs likely do not achieve long-term engraftment [2], the time that they remain at the injury site is sufficient to trigger tissue repair [26, 27].

Using a combination of RNA sequencing and quantitative mass spectrometry-based proteomics, we recently comprehensively characterized and compared the proteomes of CPCs and BM-MSCs, finding a clear overrepresentation of angiogenic-related cell surface proteins in CPCs [28]. In the present study, we further analyzed the protein composition of the plasmatic membrane fraction in CPCs and BM-MSCs in terms of interactions with other proteins or sets of molecules, in an attempt to understand the mechanisms that promote cell retention and engraftment in the heart. Plasmatic proteins identified by proteomic analysis of biotinylated fractions grouped into biological processes related to adhesion processes both in CPCs and BM-MSCs. Identified KEGG (Kyoto Encyclopedia of Genes and Genomes) pathways were commonly expressed in both cell types. However, only CPCs showed the involvement of the Rap1 signaling pathway, a key mediator of integrin-mediated cell adhesion processes. Moreover, interactome analysis of the receptome in CPCs versus BM-MSCs showed an enrichment of cell adhesion mechanisms in CPCs whereas BM-MSCs showed a robust immunoregulatory phenotype. These data improve our understanding of the mechanisms of action of CPCs and BM-MSCs in relation to cardiac repair.

## 2. Materials and Methods

### 2.1. Cells, Culture Conditions, and Lentiviral Transduction of CPCs

CPCs were obtained from Coretherapix SLU (Tigenix Group, Madrid, Spain) and were isolated as described [5]. After thawing, cells were cultured in a combination of Dulbecco's Modified Eagle's Medium/Nutrient mixture F12 (DMEM/F12) and Neurobasal medium (1 : 1), supplemented with 10% FBS ESCq (fetal bovine serum embryonic stem cell qualified), L-glutamine (2 mM), penicillin/streptomycin (P/S, 100 U/mL and 100 *μ*g/mL, respectively), B27 (0.5×), N2 supplement (0.5×), insulin-transferrin-selenium (0.5×) (all from Thermo Fisher Scientific), *β*-mercaptoethanol (50 *μ*M, Sigma-Aldrich), bFGF (10 ng/mL), IGF-II (30 ng/mL), and EGF (20 ng/mL) (all from Peprotech). Cells were grown at 37°C in humidified chamber at 3% O_2_ atmosphere.

Human BM-MSCs were purchased from Inbiomed (Inbiobank, San Sebastián, Guipuzcoa, Spain) and were expanded following the manufacturer's instructions. Briefly, cells were cultured in DMEM low-glucose (Sigma-Aldrich) supplemented with 10% FBS (Corning) and 1% P/S. Cells were grown in a humidified atmosphere of 95% air and 5% CO_2_ at 37°C.

CPCs and BM-MSCs were infected with the pSIN-EF1*α*-GFP-IRES-Puro lentiviral plasmid to label cells prior intramyocardial transplantation. Briefly, 1.7 × 10^6^ cells were seeded and incubated with 4.25 mL of virus supernatant with 8 *μ*g/mL polybrene for 2 days. Cells were then centrifuged at 1,000 × *g* for 1 h at 37°C, culture medium was replenished, and cells were incubated for 2 further days for green fluorescent protein (GFP) analysis. Transduction efficiency was measured using flow cytometry to quantify the percentage of GFP-positive cells (CPC-GFP) in transduced cells compared with the nontransduced negative control cells. GFP expression was quantified by MFI (mean fluorescence intensity) in GFP-positive cells by flow cytometry: expressed values are normalized MFI corresponding to an increasing factor of fluorescence intensity in GFP-positive transduced cells compared with GFP-negative control cells (=MFI(GFP+)/MFI(GFP−)). Expression levels acceptable for *in vivo* GFP-positive cell detection were also visually evaluated by fluorescence microscopy.

### 2.2. Animals

A total of 30 male nude rats weighing 200–250 g (HIH-Foxn1 rnu, Charles River Laboratories, Inc.) were used in the present study. Animals were randomly divided into three experimental groups (CTRL as the control, BM-MSCs, and CPCs). All procedures were approved by national and local ethical committees (reference number 2016/VSC/PEA/00006) and complied fully with the Directive 2010/63/EU of the European Parliament on the protection of animals used for scientific purposes.

### 2.3. Myocardial Infarction and Cell Transplantation

Permanent ligation of the left coronary artery was performed as described [29]. Briefly, rats were intubated and anesthetized with a mixture of O_2_/sevoflurane, and a rate of 100 cycles/min and a tidal volume of 2.5 mL (Harvard Apparatus Small Animal ventilator Model 683) and, after thoracotomy, AMI was induced by permanent ligation of the left descending coronary artery (LAD) with 6-0 prolene (Braun). The infarcted area was visualized immediately after ligation by development of a pale color in the distal myocardium. Immediately after LAD ligation, rats received an intramyocardial injection of phosphate-buffered saline (PBS) as CTRL, or a final dose of 2.5 × 10^5^ cells (BM-MSCs or CPCs) through 2 injections of 10 *μ*L at 2 points of the infarct border zone using a Hamilton syringe. Cells were administered with fluorescence microspheres at a ratio of 1 : 40 to check that the injections were performed correctly. The incision was closed with a 3-0 silk suture, and metamizole (0.4 g/mL) was given intraperitoneal (0.5 mL/kg) as pain relief. At 4-week postimplantation, rats were euthanized with an overdose of ketamine (125 mg/kg), valium (10 mg/kg), and atropine (50 mg/kg); the hearts were removed, washed with PBS, and fixed in 2% paraformaldehyde (PFA). The hearts were then embedded in paraffin and cut into 6 *μ*m slices.

### 2.4. Echocardiography

Cardiac functional assessment was determined by echocardiography as described [29], at baseline and after AMI at 4 weeks after the cell transplantation. Briefly, rats were anesthetized as before, and echocardiography was performed using an echocardiographic system (Vivid 7; GE Healthcare) with a 10 MHz linear-array transducer. Measurements were performed in M-Mode and two-dimensional (2D) images at the level of the papillary muscles. The following parameters were measured: left ventricular (LV) dimensions at end diastole (LVd) and end systole (LVs), anterior and posterior wall (AW and PW, respectively) dimensions in diastole and systole, end-diastolic area (EDA), and end-systolic area (ESA). Percentage changes in AW thickness (AWT) were calculated as %AWT = (AWs/AWd − 1) × 100. Fractional shortening (FS) was calculated as [(LVDd − LVDs)/LVDd] × 100, and fractional area change (FAC) was calculated as %FAC = [(EDA–ESA)/EDA] × 100.

### 2.5. Morphometry and Immunohistochemistry Analysis

Hearts were fixed with 2% PFA, and LV infarct size was measured in 10–12 transverse sections of 6 *μ*m (1 slice every 200 *μ*m of tissue from apex to base) stained with Masson's trichrome. Images were captured under a light Leica DMD 108 microscope. The fibrotic zone was identified by the light blue color, and scar area was determined by computer planimetry of the fibrotic regions using ImageJ software (National Institutes of Health). Infarct size was expressed as percentage of total left ventricular area and as a mean of all slices from each heart. Left ventricular wall thickness (LVW) was measured and expressed in millimeters.

### 2.6. Biodistribution Experiments

CPC-GFP cells were intramyocardically transplanted into infarcted hearts, and rats were euthanized 2, 10, and 21 days after cell transplantation. The following organs were extracted: the blood, bone marrow, spleen, heart, kidney, liver, brain, lung, and testes. Upon extraction, the tissue was placed in criovials, which were then immersed in liquid N_2_ and stored at -80°C until their processing. Genomic DNA was isolated using the Qiagen DNAeasy Tissue kit (Qiagen).

The amount of human-specific DNA in each rat organ was traced using quantitative and highly-sensitive human *Alu* sequence-specific real-time polymerase chain reaction (qPCR) analysis. The amount of human-specific DNA in each rat tissue was determined by comparing the fluorescence signal of the tested DNA with that from the positive control DNA standard using TaqMan™ Technology. Data are represented as the percentage of animals in where human-specific DNA was detected.

The organs of two animals per day of sacrifice were fixed in 2% PFA, embedded in paraffin, and processed as for morphometry and immunohistochemistry analysis for the detection of the injected GFP-labeled cells in histological sections.

### 2.7. Biotin Labeling of Surface Proteins

CPCs and BM-MSCs were surface biotinylated and processed as described [30]. Briefly, cells were incubated with 0.5 mg/mL of sulfo-NHS-SS-biotin (Thermo Fisher Scientific) for 30 min at 4°C. Then, cells were washed and lysed in 50 mM HEPES pH 7.4, 140 mM NaCl, 10% glycerol, 1% Triton X-100, 1 mM EDTA, 2 mM EGTA, and 0.5% deoxycholate. Biotin-conjugated cell surface proteins were purified with 30 *μ*L of streptavidin-agarose resin (Sigma-Aldrich). Resin was washed twice and subsequently denatured with Laemmli sample buffer for proteomic analysis.

### 2.8. Proteomic Analysis

Proteomic analyses were performed as described [31]. Briefly, 30 *μ*g of protein samples were used in 12.5% acrylamide SDS-PAGE electrophoresis and protein content was digested with trypsin. Digestion was stopped with 1% trifluoroacetic acid, and 5 *μ*L of each sample was loaded for liquid chromatography and tandem mass spectrometry (LC-MS/MS). Data were analyzed using the ProteinPilot default parameters (ProteinPilot v4.5. search engine, AB Sciex). The unused protein score, measured as the protein confidence calculated from the peptide confidence for peptides from spectra, was used to rank the proteins. Only proteins showing an unused score > 1.3 and identified with confidence ≥ 95% were included in the analysis.

Bioinformatics analyses were performed to identify functional enrichment based on overrepresentation methods implemented in the Bioconductor clusterProfiler [32] package from R (R Development Core Team 2008), using functional information from the Gene Ontology (GO) [33] and KEGG pathway databases [34].

Finally, the results were summarized and represented graphically using dotplots [32] and treemaps from the REVIGO web application [35]. Venn diagrams were created using the open source online tool Venny 2.1.0 (Oliveros, JC, CNB-CSIC. http://bioinfogp.cnb.csic.es/tools/venny/).

### 2.9. Interactome Analysis

The protein interactome was screened by matching significant proteins against the BioGRID database [36]. Gene Set Enrichment Analysis (GSEA) [37] was performed with the complete interactome, to detect significant GO biological processes, molecular functions, and cellular components.

### 2.10. Statistical Analyses

Data are represented as mean ± standard error (SE). Statistical analyses were carried out using GraphPad Prism 6 software (GraphPad Software Inc., La Jolla, CA). Statistical significance was determined using one-way ANOVA and appropriate *post hoc* analysis. Differences were considered statistically significant at *p* < 0.05 with a 95% confidence interval.

## 3. Results

### 3.1. CPC Are More Effective Than BM-MSC in Improving Cardiac Function and Reducing Infarct Size

**Figure 1:**
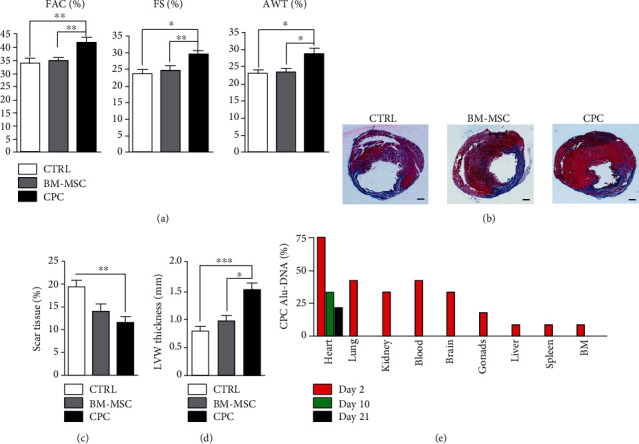
Improvement of left ventricular function in CPC-treated animals 4 weeks after transplantation. (a) Quantified values of fractional area change (FAC, %), fractional shortening (FS, %), and anterior wall thickening (AWT, %) from the control, BM-MSC, and CPC animal groups measured in 2D and M-Mode imaging 4 weeks after myocardial infarction (*n* = 10 in each group). (b) Representative images of heart sections from infarcted rats stained with Masson's trichrome. Fibrotic area in the left ventricle is stained in blue. (c) Quantification of the fibrotic area represented as the percentage scar tissue. (d) Quantification of the left ventricular wall (LVW) thickness in millimeters. Data are represented as mean ± SEM. ^∗^*p* < 0.05, ^∗∗^*p* < 0.01, and ^∗∗∗^*p* < 0.001. (e) Detection of transplanted CPCs after transplantation in infarcted rats at different time points. Percentage of rats in which human Alu-DNA was detected in the indicated organs on days: 2 (red), 10 (green), and 21 (black) after myocardial infarction. Scale bar = 1 mm.

To evaluate the effect of CPC and BM-MSC transplantation in infarcted rats, we performed echocardiographic studies at baseline and just prior to sacrifice (4 weeks) in all the experimental groups. Due to the regenerative properties attributed to BM-MSCs [11, 38], they were transplanted at the same dose of CPCs and were used as a positive control. The echocardiographic parameters analyzed in untreated animals (CTRLs) revealed a stronger deterioration of cardiac systolic function in comparison with animals treated with CPCs (Table 1). Rats subjected to cell transplantation showed a significant improvement in all echocardiography parameters measured at 4-week posttransplantation when compared with control animals: %FAC was 41.59 ± 1.92 in the CPC group, 34.76 ± 1.12 in the BM-MSC group, and 32.72 ± 1.92 in the CTRL group (*p* < 0.01) and %FS was 29.27 ± 1.08 in the CPC group, 24.51 ± 1.33 in the BM-MSC group, and 23.52 ± 1.24 in the CTRL group (*p* < 0.05). Significant differences were also observed in terms of the %AWT, with 28.60 ± 1.58 in the CPC groups, 23.22 ± 1.13 in the BM-MSC group, and 22.93 ± 0.91 in CTRL group (*p* < 0.05) (Figure 1(a)).

To examine the effect of cell transplantation on infarct size, cross-sections from the hearts of animals transplanted with CPCs, BM-MSCs, and control animals were stained with Masson's trichrome following sacrifice (Figure 1(b)). The fibrous scar tissue area was smaller in both the BM-MSC- and CPC-transplanted animals than in control animals (Figure 1(c)), although significant differences were found only between the control and CPC groups (19.15 ± 1.58 vs. 11.42 ± 1.10, respectively, *p* < 0.01). Significant differences were also found between the treatments in terms of LVW: 0.79 ± 0.07 in the control, 0.96 ± 0.10 in the BM-MSC group, and 1.51 ± 0.12 in the CPC group (Figure 1(d)); the control vs. CPCs (*p* < 0.001) and BM-MSCs vs. CPCs (*p* < 0.05).

### 3.2. Human CPCs Show Cardiac Engraftment in Rat Hearts

One of the major hurdles to the development of cell therapies is the low rate of cell survival and engraftment in the recipient heart. MSCs are used in cell therapy because of their potent immunomodulatory properties rather than their ability to differentiate and engraft the injured tissues [39]. Indeed, our previous study showed little or no retention of BM-MSCs in the heart and poorer improvement of cardiac function when suboptimal doses of cells were used (less than 10^6^ cells/animal) [38].

From the initial intramyocardial transplantation of 2.5 × 10^5^ human CPCs at the infarction border site in infarcted nude rats, we quantified the presence of human cells in different organs and in blood samples though the detection of human-specific *Alu* sequences by qPCR (Figure 1(e)). Human CPCs were detected in the hearts of 75%, 33%, and 21% of the treated nude rats at 2, 10, and 21 days after cell administration, respectively. Human cells were also observed in the blood, lungs, brain, kidney, gonads, bone marrow, liver, and spleen 2 days after cell administration. The detection of cells in these organs demonstrated that most of the infused CPCs reached the coronary circulation and were successfully distributed systemically even after intramyocardial administration. However, the number of CPCs in these organs decreased to undetectable levels at 10 days and cells were only observed in hearts in 33% of the treated rats at this time. As the CPCs were cleared in most of organs at 10 days posttransplantation, we checked for the presence of CPCs in hearts of rats sacrificed at 21 days, finding that these cells were present in the hearts of 21% of the treated rats. Moreover, an analysis of the spatial biodistribution of CPC-GFP cells by immunofluorescence in the tissue sections confirmed that the cells were found in the myocardium around the infarcted area at day 2 after cell injection and remained in the heart at least 21 days, although the number of cells decreased considerably along this time. The analysis of hearts injected with BM-MSC and sacrificed 21 days posttransplantation showed absence of GFP signal (Supplemental Figure 1).

### 3.3. CPCs and BM-MSCs Have Different Plasmatic Membrane Protein Profiles

**Figure 2:**
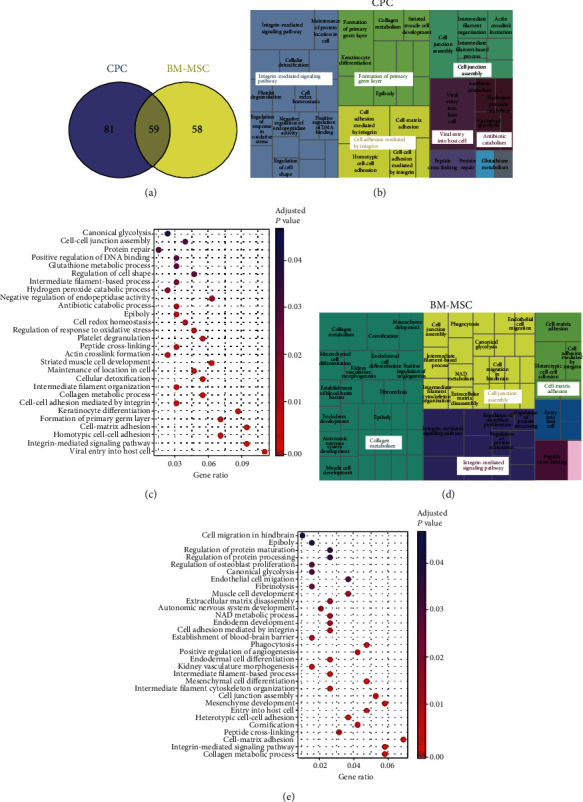
Graphical representation of upregulated GO biological processes identified by proteomic analysis in CPCs and BM-MSCs. (a) Venn diagram of data from proteomic analysis of membrane fractions, 140 proteins were expressed in CPCs, 117 were expressed in BM-MSCs, and 59 proteins were commonly expressed in both cell types. (b) Treemap diagram of biological processes overrepresented in cardiac-derived stromal cells using REVIGO webtool after proteomic analysis. (c) Dotplot representing GO biological processes overrepresented in CPCs. (d) Treemap diagram of biological processes significantly overrepresented in bone marrow mesenchymal stem cells using REVIGO webtool after proteomic analysis. (e) Dotplot representing GO biological processes significantly overrepresented in BM-MSCs.

Surface proteins are implicated in numerous cellular processes including cellular adhesion, signaling, and extracellular matrix organization. We performed comparative cell surface biotinylation to identify the most common proteins expressed on the plasmatic membrane of CPCs and BM-MSCs. Although the same amount of total protein sample was analyzed from the two groups of cells, we found significant differences in identified proteins between CPCs and BM-MSCs. Proteomic analysis of biotinylated membrane fractions showed that 59 (30%) proteins were commonly expressed between CPCs and BM-MSCs (Figure 2(a)), with many of these proteins belonging to the integrin and collagen families. Furthermore, 81 proteins (40.6%) were specifically detected in CPCs and 58 (29.4%) in BM-MSCs (Figure 2(a)). Of note, proteins implicated in cell adhesion were the most widely expressed in both cells types: 15 proteins (26.3%) related to adhesion were expressed in both cell types, 20 (35.1%) specifically in CPCs, and 22 (38.6%) in BM-MSCs. Nevertheless, the unused protein score, a measure of the protein confidence for a detected protein used to rank the proteins, showed highest values in CPCs for almost all the proteins identified. Total proteomic analysis of CPCs and BM-MSCs can be found in Supplementary Table S1 and S2, respectively.

### 3.4. CPC Plasmatic Membrane Is Significantly Enriched in Proteins Involved in Adhesion Processes

We created a treemap of significantly enriched biological processes identified in CPCs and BM-MSCs using REVIGO (Figures 2(b) and 2(d)). In this representation, each rectangle signifies a single cluster, and the clusters are joined into ‘superclusters' of loosely related terms, visualized with different colors and with the size of the rectangles reflecting the *p* value. Biotinylated proteins in the CPC group mainly were grouped into integrin-mediated signaling pathway, cell adhesion mediated by integrin, formation of primary germ layer, and cell junction assembly superclusters; the most significant processes clustered around integrin-mediated signaling pathway, cell adhesion, and cell junction assembly (Figure 2(b)). In the case of BM-MSC biotinylated proteins, GO biological processes were mostly grouped into collagen metabolism, cell junction assembly, integrin-mediated signaling pathway, and cell-matrix adhesion superclusters (Figure 2(d)). As a complementary representation, significant biological processes were depicted as dotplots, with the significance of each biological process described by its *p* value and its GeneRatio (Figures 2(c) and 2(e)), which represents the percentage of the total quantified proteins to which the function is associated. Integrin-mediated signaling pathway and cell junction assembly superclusters were also overrepresented, and most of the biological processes identified in CPCs were also represented in BM-MSCs as reflected in the dotplot graphics (Figures 2(c) and 2(e)). Total GO biological processes significantly overrepresented in CPCs and BM-MSCs are listed in Supplementary Tables S3 and S4, respectively. Similar results were obtained after KEGG pathway analysis to examine the molecular networks significantly enriched in both cell types. The most significant overrepresented KEGG pathways identified in CPCs and BM-MSCs are listed in Table 2. Of particular note was the Rap1 signaling pathway, which was exclusively overrepresented in CPCs. Rap1 signaling is involved in angiogenesis, cell adhesion, proliferation, and migration processes and could confer an additional mechanism upon CPCs to promote cell adhesion and migration.

### 3.5. Target Genes of Identified Proteins of CPC Plasmatic Membranes Are Involved in Adhesion Processes

**Figure 3:**
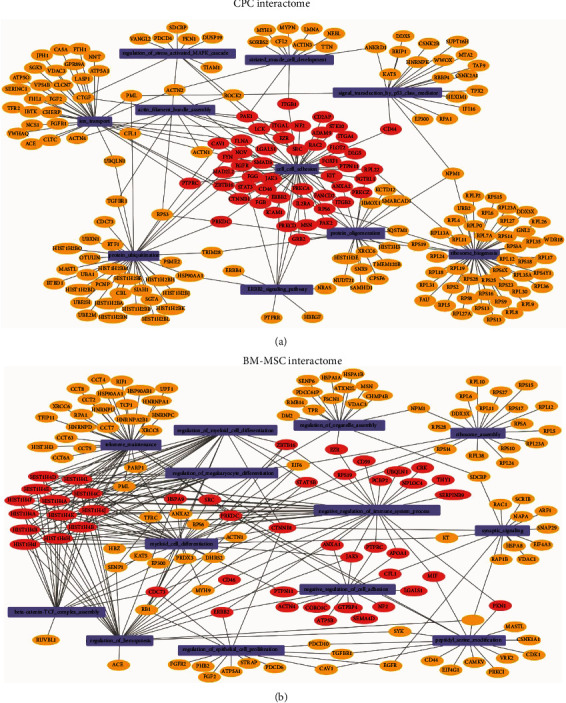
Graphical representation of CPC (a) and BM-MSC (b) interaction networks based on proteomics data sets.

We next conducted an interactome analysis in CPCs and BM-MSCs to study the target genes of proteins expressed in the plasmatic membrane and how they interact with each other. To do this, GO biological processes were identified using GSEA and visualized with Cytoscape software v3.7.2 (Figures 3(a) and 3(b)). In agreement with the aforementioned results, biological processes related to adhesion mechanisms were enriched in the plasmatic membrane of CPCs, whereas the protein repertoire overexpressed in the plasmatic membrane of BM-MSCs was more related to immunological processes (Supplemental Tables 5 and 6).

## 4. Discussion

According to the World Health Organization, 32.4 million cases of myocardial infarction or stroke occur worldwide every year. Because current therapies diminish disease progression without contributing significantly to repair, cardiac regeneration is a major therapeutic objective in cardiology. Both CPCs and MSCs have demonstrated their potential to regenerate infarcted hearts after AMI in different animal models [5, 26, 29, 38, 40], and their beneficial effect on left ventricular function has been widely demonstrated in spite of their low engraftment and retention after infusion [4, 41].

Here, we show the superior capacity of CPCs over BM-MSCs in improving cardiac functional parameters and reducing infarct size in a rat model of induced MI. BM-MSCs have been extensively used in preclinical studies of cardiac regeneration; however, in accord with previous studies [27], CPCs seem to be significantly more effective at improving functional cardiac parameters at lower doses than those used with BM-MSC. At the doses assessed in this study, improvement of cardiac function was evident when CPC transplantation was compared with controls measured in changes of FAC, FS, and AWT. We observed similar results in terms of viable myocardium, with only CPCs producing significant improvements in the reduction of scar tissue and increase of LVW thickness. BM-MSCs did show a positive trend in some of these parameters, but they were not statistically significant, likely due to the low cell dose used. These results suggest that although both cell types are able to promote functional improvements in all measured parameters the effects on cardiac repair are more pronounced using CPCs.

Given the relevant role of plasmatic membrane receptors in extravasation and infiltration, we sought to better characterize the protein expression profiles in the plasmatic membrane of CPCs and BM-MSCs, to inform on the mechanisms that might favour cardiac regeneration. It has been described previously that CPC highly engraft in infarcted hearts [2, 4]. Accordingly, the proteins putatively implicated in the superior engraftment and retention in the heart of CPCs relative to BM-MSCs were studied by proteomic analysis of the biotinylated surface proteins.

Comparative proteomic analysis of CPC versus BM-MSC membrane proteins revealed a larger number of proteins corresponding to the collagen and integrin families commonly expressed in CPCs. Integrins are a family of cell adhesion receptors widely described to bind a broad variety of ligands and cell surface adhesion proteins [42]. Interestingly, most of the integrin heterodimers described to be collagen-, fibronectin-, and laminin-binding receptors [43] were identified in CPCs, which might explain the stronger capacity of CPCs to remain and be engrafted. We identified the integrin subunits *α*2, *α*3, *α*5, *β*1, *β*3, and *β*5, which form part of the collagen, vitronectin, and fibronectin receptors. In addition, collagen subunits *α*1, *α*2, and *α*3, and also different proteins implicated in cell adhesion such as fibulin-1, catenin *β*1, nectin-2, and cadherin-13 were also overexpressed in CPCs versus BM-MSCs, in accordance with previous results [23, 28]. Some proteins identified in the plasmatic membrane of BM-MSCs including integrin *α*V, *β*1, *α*11, and *α*5 act as adhesion molecules [44]. Thus, our data indicate that, as expected, CPCs and BM-MSCs express plasma proteins involved in the regulation of cell adhesion, but the richer profile in CPCs suggests a more integrated regulatory network involved in cell adhesion, which rationalizes their stronger retention potential. As a matter of fact, no heart-specific adhesion molecule did significantly show up in our analysis indicating that CPC's adhesion capacity is not tissue specific but wide ranging. These features might be behind the fact that we have been able to detect only CPC in different tissues and not BM-MSCs. It has been previously described that BM-MSCs are retained and detected in different tissues including the heart [45] but the cell dose used in those studies is higher than the one used in this study which might explain the discrepancy.

Identification of regulated pathways by KEGG pathway enrichment analysis revealed extracellular matrix-receptor interaction, focal adhesion, and cell adhesion molecules among the most significantly overrepresented processes in both CPCs and BM-MSCs. Of note is the selective expression in CPCs of the Rap1 signaling pathway, a key mediator of integrin-mediated cell adhesion processes. Rap1, a member of the Ras superfamily of small GTP-binding proteins, is a conserved regulator of cardiovascular signaling. Several essential functions are directly regulated by Rap1 signaling, including cellular adhesion, cell proliferation, and formation of cell junctions mediated by connexion-43 [46, 47]. In addition, Rap1 regulates processes at the plasmatic membrane and integrin-mediated cell adhesion [48]. These results suggest that Rap1 could be involved in the mechanisms that promote the cellular adhesion/engraftment of CPCs to the heart. According to our analysis and taking into account the literature, we consider that our work reinforces the idea that CPCs, enhanced engraftment is responsible of the healing benefits and that Rap1 signaling pathway is an important regulator of the engraftment. Further studies will be performed to better understand the exact role of the Rap1 on the observed phenotype.

While most of the proteins were commonly expressed in both cell types, the interactome analysis showed that GO biological processes upregulated in CPCs were mostly related with adhesion, whereas the upregulated functions in BM-MSCs were mainly related to immune regulation processes. With regard to BM-MSCs, our findings are in general agreement with current knowledge on the biological processes that occur immediately after AMI, including the mobilization of innate and adaptive immune cells including monocytes, neutrophils, mast cells, and macrophages [25, 49] and the well-known ability of BM-MSCs to modulate the immune response [50, 51] and to target immune cells [21].

In summary, our study demonstrates that the plasmatic membrane of CPC cells contains proteins actively implicated in biological processes related to cellular adhesion mechanisms. CPCs strongly engraft to the heart after intramyocardial injection and significantly improve cardiac function. Thus, these findings support the further evaluation and development of CPCs as strong candidates for cell therapy in cardiac repair.

## 5. Conclusions

Taking all together, our results indicate that CPC cells are able to improve cardiac function and promote tissue repair after myocardial infarction due to a stronger capacity to engraft in the infarcted area. Our data deepens in the mechanistic differences between CPCs and BM-MSCs and suggests that both cell types might be complementary as a therapeutic strategy.

## Figures and Tables

**Table 1:** Echocardiographic values of the control, BM-MSC, and CPC groups at baseline and 4 weeks after myocardial infarction.

	CTRL		BM-MSC		CPC		*p* values^∗^			
	(*n* = 10)		(*n* = 10)		(*n* = 10)		ANOVA	Control vs. BM-MSC	Control vs. CPC	BM-MSC vs. CPC
	Baseline	Final^∗^	Baseline	Final^∗^	Baseline	Final^∗^				
AWd	1.61 ± 0.03	1.06 ± 0.03	1.29 ± 0.02	0.97 ± 0.03	1.46 ± 0.01	1.03 ± 0.04		0.0031		
LVd	5.86 ± 0.07	7.43 ± 0.17	4.87 ± 0.21	6.47 ± 0.18	5.92 ± 0.08	7.14 ± 0.16	0.0154	0.0031		0.0381
PWd	1.49 ± 0.07	1.53 ± 0.08	1.38 ± 0.06	1.46 ± 0.14	1.41 ± 0.04	1.33 ± 0.04			0.0495	
AWs	2.45 ± 0.05	1.37 ± 0.04	2.10 ± 0.08	1.29 ± 0.05	2.30 ± 0.03	1.43 ± 0.06				
LVs	3.39 ± 0.05	5.68 ± 0.17	2.91 ± 0.12	5.00 ± 0.23	3.50 ± 0.04	5.10 ± 0.14	0.0407		0.0251	
PWs	2.10 ± 0.08	2.04 ± 0.12	1.86 ± 0.10	2.07 ± 0.13	2.12 ± 0.07	1.87 ± 0.06				
EDA	31.48 ± 0.62	43.90 ± 1.59	26.86 ± 1.70	37.86 ± 2.11	31.15 ± 0.55	43.35 ± 1.75				
ESA	8.82 ± 0.39	29.36 ± 1.42	7.46 ± 0.55	24.05 ± 1.36	8.27 ± 0.21	25.49 ± 1.44		0.0196		
FS	71.99 ± 1.08	33.28 ± 1.42	71.04 ± 1.09	36.85 ± 2.06	73.42 ± 0.54	41.37 ± 1.85	0.0065		0.0024	
FAC	42.11 ± 0.46	23.67 ± 0.88	40.75 ± 0.88	26.49 ± 1.80	40.90 ± 0.50	28.60 ± 0.93	0.0072		0.0008	
AWT	33.96 ± 0.90	22.57 ± 0.75	37.96 ± 1.21	23.81 ± 1.03	36.55 ± 0.74	27.65 ± 1.00	0.0081		0.0020	

Abbreviations: AWd: anterior wall diastole thickness; AWs: anterior wall systole thickness; AWT: anterior wall thickening; EDA: end-diastolic area; ESA: end-systolic area; FAC: fractional area change; FS: fractional shortening; LVd: left ventricular diastole internal dimension; LVs: left ventricular systole internal dimension; BM-MSC: bone marrow mesenchymal stem cells; PWd: posterior wall diastole thickness; PWs: posterior wall systole thickness; w: weeks. All values are mean ± SEM. AWd, LVd, PWd, AWs, LVs, and PWs are expressed in mm whereas EDA and ESA are expressed in mm^2^. FS, FAC, and AWT are expressed as percentage.

**Table:** (a) Cardiac progenitor/stem cells

KEEG_id	Term	*p* value
hsa04512	ECM-receptor interaction	2.22*E* − 17
hsa04510	Focal adhesion	4.06*E* − 14
hsa05412	Arrhythmogenic right ventricular cardiomyopathy (ARVC)	1.74*E* − 12
hsa05165	Human papillomavirus infection	1.74*E* − 10
hsa05410	Hypertrophic cardiomyopathy (HCM)	4.29*E* − 10
hsa05414	Dilated cardiomyopathy (DCM)	6.81*E* − 10
hsa05205	Proteoglycans in cancer	2.25*E* − 08
hsa04640	Hematopoietic cell lineage	9.61*E* − 08
hsa04810	Regulation of actin cytoskeleton	5.04*E* − 07
hsa04145	Phagosome	1.68*E* − 06
hsa04514	Cell adhesion molecules (CAMs)	2.09*E* − 05
hsa04670	Leukocyte transendothelial migration	3.92*E* − 05
hsa05131	Shigellosis	1.16*E* − 04
hsa05100	Bacterial invasion of epithelial cells	1.62*E* − 04
hsa04974	Protein digestion and absorption	1.94*E* − 04
hsa05222	Small cell lung cancer	3.05*E* − 04
hsa05418	Fluid shear stress and atherosclerosis	1.35*E* − 03
hsa04520	Adherens junction	2.68*E* − 03
hsa04015	Rap1 signaling pathway	5.30*E* − 03
hsa04611	Platelet activation	8.31*E* − 03
hsa04919	Thyroid hormone signaling pathway	8.31*E* − 03
hsa05144	Malaria	1.28*E* − 02
hsa05130	Pathogenic Escherichia coli infection	1.70*E* − 02
hsa05206	MicroRNAs in cancer	1.78*E* − 02

**Table:** (b) Bone marrow mesenchymal stem cells

KEEG_id	Term	*p* value
hsa04512	ECM-receptor interaction	2.42*E* − 19
hsa04510	Focal adhesion	4.06*E* − 14
hsa05165	Human papillomavirus infection	7.93*E* − 12
hsa05412	Arrhythmogenic right ventricular cardiomyopathy (ARVC)	9.52*E* − 11
hsa05410	Hypertrophic cardiomyopathy (HCM)	1.60*E* − 08
hsa05205	Proteoglycans in cancer	2.25*E* − 08
hsa05414	Dilated cardiomyopathy (DCM)	2.38*E* − 08
hsa04810	Regulation of actin cytoskeleton	5.04*E* − 07
hsa04514	Cell adhesion molecules (CAMs)	1.34*E* − 06
hsa04670	Leukocyte transendothelial migration	2.03*E* − 06
hsa05100	Bacterial invasion of epithelial cells	7.42*E* − 06
hsa05131	Shigellosis	1.16*E* − 04
hsa05146	Amoebiasis	3.30*E* − 04
hsa04933	AGE-RAGE signaling pathway in diabetic complications	5.61*E* − 04
hsa04640	Hematopoietic cell lineage	6.40*E* − 04
hsa05206	MicroRNAs in cancer	2.16*E* − 03
hsa04520	Adherens junction	2.68*E* − 03
hsa04145	Phagosome	3.04*E* − 03
hsa05222	Small cell lung cancer	4.28*E* − 03
hsa04611	Platelet activation	8.31*E* − 03
hsa04919	Thyroid hormone signaling pathway	8.31*E* − 03
hsa05135	Yersinia infection	8.82*E* − 03
hsa05418	Fluid shear stress and atherosclerosis	1.27*E* − 02
hsa05144	Malaria	1.28*E* − 02
hsa05130	Pathogenic Escherichia coli infection	1.70*E* − 02

## Data Availability

The data that support the findings of this study are available from the corresponding author upon reasonable request.

## Supplementary Materials

Supplementary 1 Supplemental Figure 1. Representative fluorescence heart images of GFP-CPCs and GFP-BM-MSCs 2, 10, and 21 days postimplantation. Red fluorescent microspheres were coinjected with the cells to localize the administration sites. Images were acquired at 10x magnification. Scale bar = 100 *μ*m

Supplementary 2 Supplemental Table 1. Protein identification of biotinylated proteins from the plasmatic membrane fractions of cardiac progenitor/stem cells.

Supplementary 3 Supplemental Table 2. Protein identification of biotinylated proteins from the plasmatic membrane fractions of bone marrow mesenchymal stem cells.

Supplementary 4 Supplemental Table 3. GO biological processes of biotinylated proteins identified in the plasmatic membrane of cardiac progenitor/stem cells.

Supplementary 5 Supplemental Table 4. GO biological processes of biotinylated proteins identified in the plasmatic membrane of bone marrow mesenchymal stem cells.

Supplementary 6 Supplemental Table 5. GO biological processes were identified from the *interactome* analysis of cardiac progenitor/stem cells.

Supplementary 7 Supplemental Table 6. GO biological processes were identified from the *interactome* analysis of bone marrow mesenchymal stem cells.
