# Corrigendum to “Plasmatic Membrane Expression of Adhesion Molecules in Human Cardiac Progenitor/Stem Cells Might Explain Their Superior Cell Engraftment after Cell Transplantation”

**DOI:** 10.1155/2022/9786160

**Published:** 2022-03-04

**Authors:** Imelda Ontoria-Oviedo, Itziar Palacios, Joaquín Panadero, Belén Sánchez, Francisco García-García, Adolfo López-Cerdán, Akaitz Dorronsoro, Delia Castellano, Luis Rodríguez-Borlado, Antonio Bernad, Pilar Sepúlveda

**Affiliations:** ^1^Regenerative Medicine and Heart Transplantation Unit, Instituto de Investigación Sanitaria La Fe, 46026 Valencia, Spain; ^2^Coretherapix SLU, Tres Cantos, 28760 Madrid, Spain; ^3^IGENOMIX S. L, 46980 Paterna, Valencia, Spain; ^4^Bioinformatics and Biostatistics Unit, Centro de Investigación Príncipe Felipe, 46012 Valencia, Spain; ^5^Biomedical Imaging Unit FISABIO-CIPF, Centro de Investigación Príncipe Felipe, 46012 Valencia, Spain; ^6^Department of Immunology and Oncology, Centro Nacional de Biotecnología (CNB-CSIC), 28048 Madrid, Spain

In the article titled “Plasmatic Membrane Expression of Adhesion Molecules in Human Cardiac Progenitor/Stem Cells Might Explain Their Superior Cell Engraftment after Cell Transplantation” [1], the terms FS and FAC were inadvertently exchanged in Table 1. The authors explained that this was due to an error during the preparation of the manuscript and the corrected Table 1 is shown below:

The authors and the Editorial board agree to the publication of the corrigendum.

## Figures and Tables

**Table 1:** Echocardiographic values of the control, BM-MSC, and CPC groups at baseline and 4 weeks after myocardial infarction.

	CTRL		BM-MSC		CPC		*p* values^∗^			
	(*n* = 10)		(*n* = 10)		(*n* = 10)		ANOVA	Control vs. BM-MSC	Control vs. CPC	BM-MSC vs. CPC
	Baseline	Final^∗^	Baseline	Final^∗^	Baseline	Final^∗^				
AWd	1.55 ± 0.02	1.05 ± 0.04	1.48 ± 0.08	1.01 ± 0.02	1.47 ± 0.02	1.02 ± 0.03				
LVd	5.79 ± 0.06	7.43 ± 0.24	5.36 ± 0.22	6.94 ± 0.18	5.94 ± 0.10	7.02 ± 0.21				
PWd	1.46 ± 0.04	1.62 ± 0.06	1.45 ± 0.09	1.40 ± 0.10	1.42 ± 0.04	1.33 ± 0.05	0.0441		0.0035	
AWs	2.36 ± 0.04	1.37 ± 0.06	2.32 ± 0.11	1.33 ± 0.03	2.32 ± 0.04	1.45 ± 0.09				
LVs	3.35 ± 0.04	5.70 ± 0.24	3.15 ± 0.13	5.25 ± 0.18	3.48 ± 0.05	4.99 ± 0.15			0.0174	
PWs	2.02 ± 0.05	2.08 ± 0.11	2.03 ± 0.05	2.01 ± 0.10	2.11 ± 0.10	1.83 ± 0.08				
EDA	31.28 ± 0.76	45.10 ± 1.74	29.09 ± 1.25	39.40 ± 1.73	30.79 ± 0.70	42.64 ± 2.27				
ESA	8.55 ± 0.51	30.04 ± 1.85	8.44 ± 0.43	26.09 ± 1.10	8.10 ± 0.29	25.76 ± 1.68				
FAC	72.64 ± 1.47	32.72 ± 1.92	71.40 ± 0.92	34.76 ± 1.12	73.69 ± 0.66	41.59 ± 1.92	0.0114		0.0024	0.0089
FS	42.13 ± 0.56	23.52 ± 1.24	40.90 ± 0.76	24.51 ± 1.33	41.37 ± 0.64	29.27 ± 1.08	0.0103		0.0039	0.0232
AWT	34.14 ± 1.19	22.93 ± 0.91	36.35 ± 1.43	23.22 ± 1.13	36.63 ± 1.07	28.60 ± 1.58	0.0219		0.0173	0.0351

Abbreviations: AWd: anterior wall diastole thickness; AWs: anterior wall systole thickness; AWT: anterior wall thickening; CPCs: cardiac progenitor/stem cells; CTRL: control; EDA: end-diastolic area; ESA: end-systolic area; FAC: fractional area change; FS: fractional shortening; LVd: left ventricular diastole internal dimension; LVs: left ventricular systole internal dimension; BM-MSCs: bone marrow mesenchymal stem cells; PWd: posterior wall diastole thickness; PWs: posterior wall systole thickness. All values are mean ± SEM. AWd, LVd, PWd, AWs, LVs, and PWs are expressed in mm, whereas EDA and ESA are expressed in mm^2^. FS, FAC, and AWT are expressed as percentage.

## References

[1] Ontoria-Oviedo I., Palacios I., Panadero J. (2020). Plasmatic membrane expression of adhesion molecules in human cardiac progenitor/stem cells might explain their superior cell engraftment after cell transplantation. *Stem Cells International*.

