# Supplementary figures and images for: Plasmatic Membrane Expression of Adhesion Molecules in Human Cardiac Progenitor/Stem Cells Might Explain Their Superior Cell Engraftment after Cell Transplantation
Source: Stem Cells Int. 2020 Oct 10;2020:8872009. doi: 10.1155/2020/8872009 (PMC7569451; doi:10.1155/2020/8872009)

CPC

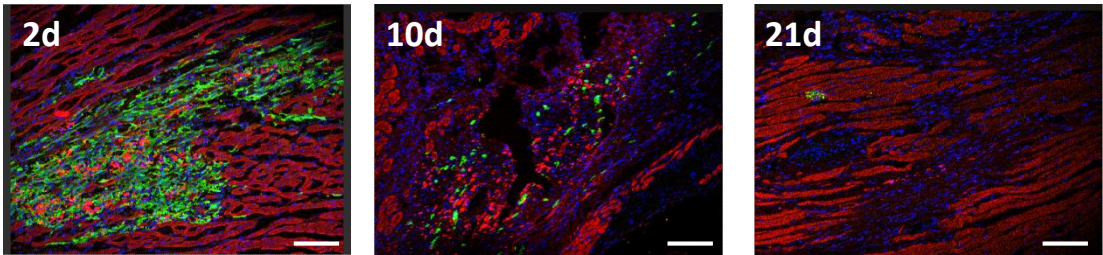

BM-MSC

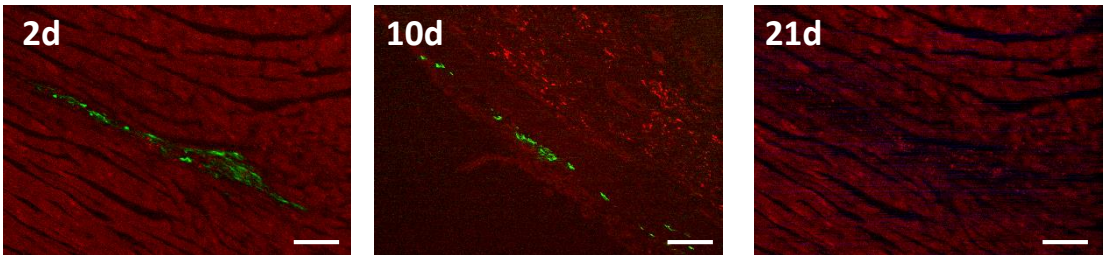

Supplement: Supplementary 1 — Supplemental Figure 1. Representative fluorescence heart images of GFP-CPCs and GFP-BM-MSCs 2, 10, and 21 days postimplantation. Red fluorescent microspheres were coinjected with the cells to localize the administration sites. Images were acquired at 10x magnification. Scale bar = 100 μm [file 8872009.f1.pdf]
